# Effect of soil management systems on the rhizosphere bacterial community structure of tobacco: Continuous cropping vs. paddy-upland rotation

**DOI:** 10.3389/fpls.2022.996858

**Published:** 2022-09-20

**Authors:** Peng Wang, Shen Yan, Wenshui Zhang, Xiaodan Xie, Mingjie Li, Tianbao Ren, Li Gu, Zhongyi Zhang

**Affiliations:** ^1^College of Agriculture, Fujian Agriculture and Forestry University, Fuzhou, China; ^2^Fujian Tobacco Monopoly Bureau, Fuzhou, China; ^3^Staff Development Institute of China National Tobacco Corporation, Zhengzhou, China; ^4^College of Tobacco, Henan Agricultural University, Zhengzhou, China

**Keywords:** rhizosphere, microbial community structure, tobacco, continuous cropping, rotation cropping

## Abstract

Rhizosphere bacteria play important role in soil nutrient cycling and plant growth, and their richness and diversity are influenced by soil management systems. However, the specific changes in tobacco rhizosphere bacterial community structure in continuous and tobacco-rice rotation cropping systems remain uninvestigated. In this study, soil properties and the composition of the rhizosphere bacterial community in tobacco monocropping and tobacco-rice rotation cropping systems were analyzed. Moreover, the comparison of rhizosphere bacterial community structure between tobacco continuous and tobacco-rice rotation cropping systems was performed *via* high-throughput sequencing. The changes in the composition of the rhizosphere bacterial community were investigated at different tobacco growth stages. The results showed that continuous tobacco cropping increased the soil soluble organic carbon (SOC), total nitrogen (TN), and the content of other nutrients (e.g., available phosphorus and available potassium) compared to tobacco-rice rotation cropping. However, monocropping decreased bacterial alpha-diversity and altered the community composition when compared to the rotation cropping system. At the phylum level, the relative abundance of Proteobacteria, Gemmatimonadetes, and Bacteroidetes increased in the continuous cropping soil, while that of Acidobacteria, Firmicutes, and Actinobacteria decreased. At the genera level, the average abundance of the dominant genus *Bacillus* varied from 12.96% in continuous cropping libraries to 6.33% in the rotation cropping libraries (*p* < 0.05). Additionally, several other taxa, such as *o_Acidobacteriales* and *Candidatus_Solibacter* decreased from 7.63 to 6.62% (*p* < 0.05) and 4.52 to 2.91% (*p* < 0.05), respectively. However, the relative abundance of *f_Gemmatimonadaceae* and *c_Subgroup_6* showed an increase of 1.46% (*p* < 0.05) and 1.63% (*p* < 0.05) in the tobacco-rice rotation cropping system, respectively. The results of NMDS indicated that the rhizobacteria community structure differed in the two cropping systems. In tobacco, the rhizosphere bacterial community structure showed no significant changes in the prosperous long-term stage and topping stage, but the composition changed significantly in the mature stage.

## Introduction

Tobacco is one of the most important economic crops that is mainly produced in China, India, Brazil, and Zimbabwe (Reichert et al., 2019). In China, tobacco planting is concentrated in the southwest region (Wang et al., 2019b). With the fast development of the tobacco industry and limited arable land, it is common to see the adoption of a continuous cropping system in tobacco cultivation. Many studies have confirmed that continuous cropping could lead to a reduction in yield or even the death of plants (Zhou and Ma, 2003; Jia et al., 2010). Thus, crop rotation is encouraged, and it presented certain advantages to improve the profitability of tobacco agriculture (dos Santos Canalli et al., 2020). As reported, many factors, including climate, soil properties, and soil microbes, affected the tobacco quality (Meng et al., 2015). Among these factors, soil microbes had been verified as one of the important factors that influence tobacco growth. However, the soil microbial community changes in tobacco continuous and rotation cropping systems remain to be investigated.

Soil microorganisms are critical to soil biological, chemical, and physical processes that are closely associated with soil quality, soil fertility, and productivity (Blagodatskaya and Kuzyakov, 2013; Beckers et al., 2017). The rhizosphere soil microbes affect the absorption and transformation of soil nutrients (Fierer et al., 2012). It means that the richness and diversity of rhizosphere soil microorganisms are important factors that affect the growth, development, and health status of plants (Bron et al., 2012). Simultaneously, the plant root exudates can also determine the rhizosphere microbes and influence the microbial community composition (Doornbos et al., 2012). The continuous cropping of a single specie often leads to an imbalance in the soil microbial flora (Fu et al., 2017). In comparison, the rotation cropping of different plant species is beneficial to maintain the balance of the rhizosphere soil microbial community (Liu et al., 2022). The soil microbial community can serve as a sensitive bioindicator of soil health due to its quick response to environmental changes and close relationship with soil conditions and land management (Sharma et al., 2011), which would consequently influence plant growth and health (Gao et al., 2021). Therefore, it is necessary to evaluate the relationship between the structural changes in microbial communities and tobacco cropping systems.

As the important decomposer in soil, bacterial community composition and diversity showed significant influence on soil and ecosystem balance and plant quality (Wu et al., 2022). Studies have shown that bacterial community succession patterns and the structure of soil microbes provide important suggestions for promoting tobacco growth and improving tobacco quality. Niu et al. (2016), found that both the fallow stage and tobacco shaped the shift pattern in soil bacterial communities during tobacco cultivation under different rotation systems. Wang et al. (2018) explored the influence of nitrogen addition on the structure of bacterial communities in rhizosphere soil under continuous cropping. However, there was limited research investigating the changes in the tobacco rhizosphere bacterial community and their relationship with soil properties in continuous and rotation cropping systems. In addition, the evolution of the tobacco rhizosphere bacterial community during different growth stages would deeply comprehend the tobacco growth process.

Therefore, this research aims to (1) analyze the relationship between rhizosphere bacterial community composition and soil properties in tobacco continuous and rotation cropping systems, (2) investigate the varieties of bacterial community richness and diversity in tobacco continuous and rotation cropping systems, and (3) indicate the influence of tobacco growth stage on rhizosphere bacterial community.

## Materials and methods

### Site description

The experiment was conducted from 2019 to 2021 at Nanping city Fujian province (27°37.407′N, 118°00.159′E, and 165 m altitude). The study region is characterized by a central subtropical monsoon with the average temperature and annual precipitation of 18.08°C and 1660 mm, respectively. The test soil was fertile and supplemented with limestone, and the basic physicochemical properties are as follows: the pH of 4.86, dissolved organic carbon of 38.11 g/kg, total nitrogen of 156.24 mg/kg, available phosphorus of 46.33 mg/kg, and available potassium of 154.29 mg/kg. The test tobacco strain was “Cuibi No. 1” (CB-1), and the rotation rice was Indica-japonica hybrid rice variety Yongyou 1540.

### Field experiment design

In this study, a split plot arrangement was used, and the cropping system was the main plot treatment. The sub-plot had a width of 6 m and a length of 20 m (120 m^2^). A 0.5-m isolation zone was set up in different plots, and each experiment plot had three replications. Two cropping systems were performed in this study: tobacco continuous cropping (C) and tobacco-rice rotation cropping (R). The planting management method of all the experimental plots was the same. Tobacco was sown at a density of 16,500 plants per hm^2^. The row and plant spaces were 0.50 and 0.57 m, respectively. The field growth period of tobacco was 163 days, and 1 year was thought of as a cycle to investigate different cropping systems. Moreover, the sowing time of tobacco and rice was 4 November 2020 and 20 June 2021.

### Soil sample collection

Soil samples were collected from the tobacco rhizosphere region in the second cycle, and they were taken at the prosperous long-term stage (E), topping stage (L), and mature stage (M), respectively. In each treatment plot, three tobacco plants were selected randomly, and the rhizosphere soil was taken based on the method described previously (Sun et al., 2009). In brief, the sampling shovel was used to excavate the entire root of tobacco, and the soil that tightly adhered to the root was collected, discarding those particles that dropped naturally. Then, the samples were passed through a 2-mm sieve to remove roots, rocks, and litter. The sieved samples were divided into two parts: one portion was kept at −80^°^C for microbial community analysis and the other one was stored at 4^°^C for the analysis of basic soil physicochemical properties.

### Analysis of soil physicochemical properties

Soil and water suspension in the ratio of 1:2.5 (w:v) was taken to measure the pH value using a digital pH meter. The soluble organic carbon (SOC) and total nitrogen (TN) levels were determined by using a TOC/TN analyzer (TNC-200, Toray Engineering D Solutions Co., Ltd., Japan) after pretreatment. Briefly, 5 g of soil was extracted with 50 mL of 2 M KCl on a shaker with a speed of 200 r/min for 1 h. Then, the extracts were filtered, and the filtrate was collected and analyzed using a TOC/TN analyzer. In addition, the filtrate was used to determine the water-soluble chlorine content with the silver nitrate titration method (Tong et al., 2022). Spectrophotometry and flame photometry were used to detect soil available phosphorus (AP) and potassium (AK) after they were extracted with ammonium lactate solution (Tian et al., 2021). Moreover, the exchangeable (mild acid extractable) element portions (calcium and magnesium) were determined by extracting the soil samples with 0.11 mol/L acetic acid at a ratio of 1:20 (w/v) for 16 h (Zhao et al., 2021). Then, the soil extracts were analyzed by flame atomic absorption spectroscopy (FAAS, VARIAN SpectrAA-280, Victoria, Australia).

### DNA extraction and Illumina sequencing

The genomic DNA was extracted from 0.5 g of soil using the Fast DNA Spin Kit for Soil (MP Biomedicals, United States) based on the manufacturer’s instructions. The purity of DNA was checked by NanoDrop spectrophotometer ND-2000c (Thermo Fisher Scientific, United States). The V3-V4 regions of 16S rRNA were amplified with the primers 338F and 806R to demonstrate the composition and structure of the bacterial community (Xu et al., 2020). About 20 μl of the reaction system was applied, including 4 μL of × 5 FastPfu Buffer, 2 μL of 2.5 mM dNTPs, 0.8 μL of forward primer, 0.8 μL of reverse primer, 0.4 μL of FastPfu polymerase, 0.2 μL of BSA, 10 ng of DNA template, and ddH_2_O. After purification and library construction, the amplicons were sequenced on an Illumina HiSeq platform (Illumina, San Diego, United States).

### Statistical analysis

The independent *t*-test and the Student–Newman–Keuls test for multiple comparisons were used to assess data differences between the treatments *via* SPSS 22.0. The one-way ANOVA and “vegan” package of R software were performed to evaluate the alpha-diversity and beta-diversity. Figures were generated using Origin 2021.

## Results

### Soil characteristics

Tobacco planting significantly influenced the soil properties, and different cropping systems presented different effects. The changes in the soil properties of tobacco continuous and tobacco-rice rotation cropping systems are presented in Table 1. The pH value of tobacco continuous cropping system soil was significantly lower than that of tobacco-rice rotation cropping system soil but higher than that of the original soil. Obviously, tobacco cropping improved the acidity of the soil. Moreover, the content of SOC and TN in the tobacco continuous cropping soil was 1.58 and 1.62 times that observed in tobacco-rice rotation cropping soil, respectively. When compared to the original soil, in tobacco cropping soil, the nutritional indicators AP and AK increased by 0.51–1.03 and 1.64–3.42 times, respectively. The concentrations of AP and AK in the tobacco continuous cropping system were 1.34 and 1.67 times that observed in the tobacco-rice rotation cropping system, indicating a higher promotion in the accumulation of soil nutrients. In addition, tobacco planting significantly increased the exchangeable Ca/Mg, while it decreased the water-soluble Cl content. In both cropping systems, exchangeable Ca increased from 0.88 to 1.27 times that of the original soil, and the tobacco continuous cropping system presented a stronger influence than the tobacco-rice rotation cropping system. In short, compared with tobacco-rice rotation, the continuous cropping significantly increased soil pH, SOC, TN, AP, AK, and exchangeable Ca/Mg, and significantly decreased the soil water-soluble Cl content.

**TABLE 1:** Soil physicochemical properties of different cropping systems.

Treatment	pH	SOC	TN	AP	AK	Water-soluble Cl^–^	Exchangeable Ca	Exchangeable Mg
		(g/kg)	(mg/kg)	(mg/kg)	(mg/kg)	(mg/kg)	(mg/kg)	(mg/kg)
Original soil	4.868 ± 0.02 a	38.11 ± 5.28 a	156.24 ± 6.21 b	46.33 ± 3.87 a	154.29 ± 6.85 a	35.36 ± 3.28 c	374.98 ± 29.64 a	74.22 ± 8.62 a
C	5.58 ± 0.02 b	43.84 ± 6.14 c	184.86 ± 5.85 c	93.90 ± 4.81 c	682.35 ± 25.43 c	14.63 ± 2.54 b	706.12 ± 45.21 b	213.65 ± 12.36 c
R	5.87 ± 0.01 c	27.82 ± 5.35 b	114.32 ± 5.32 a	69.98 ± 2.54 b	407.51 ± 31.24 b	12.68 ± 3.25 a	851.69 ± 51.86 c	123.34 ± 9.52 b

SOC, soluble organic carbon; TN, total nitrogen; AP, available phosphorus; AK, available phosphorus and potassium. Different letters indicates significant differences.

### Alpha-diversity of soil microbial community

**FIGURE 1:**
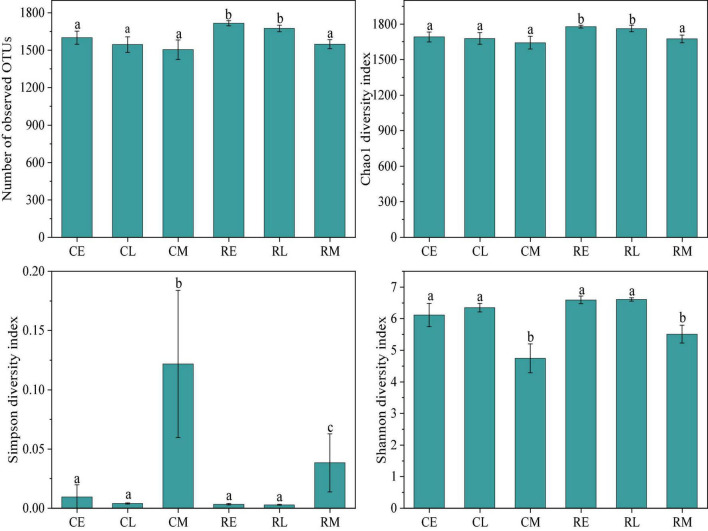
Bacterial alpha-diversity index in different groups (CE, CL, and CM represent the tobacco prosperous long-term, topping, and mature stages, respectively, in the tobacco continuous cropping system; RE, RL, and RM represent the corresponding stage in tobacco-rice rotation cropping system).

A total of 2,878,961 reads were obtained after high-throughput sequencing, and each sample had approximately 65,416 clean tags. Alpha-diversity was used to evaluate the species richness and evenness in a community (Willis, 2019). The alpha-diversity was measured using many indexes, such as Chao1, Simpson, and Shannon index (Moreno et al., 2006). As shown in Figure 1 the observed OTUs, Chao 1, Simpson, and Shannon diversity indexes presented significant differences in different cropping systems and tobacco growth stages. The observed average number of OTUs in the tobacco continuous cropping system was 1,550, which was significantly lower (*p* < 0.05) than that in the tobacco-rice rotation cropping system which showed 1,646 OTUs. In addition, the Chao1 and Shannon diversity indexes showed the same results. Moreover, the Simpson diversity index in the continuous cropping system was significantly higher (*p* < 0.05) than in the rotation cropping system, particularly during the tobacco mature period. With regard to the influence of the tobacco growth stage on the alpha-diversity of rhizosphere bacteria, the mature stage exhibited notable effects when compared to prosperous long-term and topping stages. Except for the Simpson diversity index, other alpha indexes were the lowest in the mature stage. However, there was no significant difference in the bacterial alpha-diversity values in the mature stage between different cropping systems.

### Variation in bacterial community structure

#### Bacterial community composition at different taxonomic levels

**FIGURE 2:**
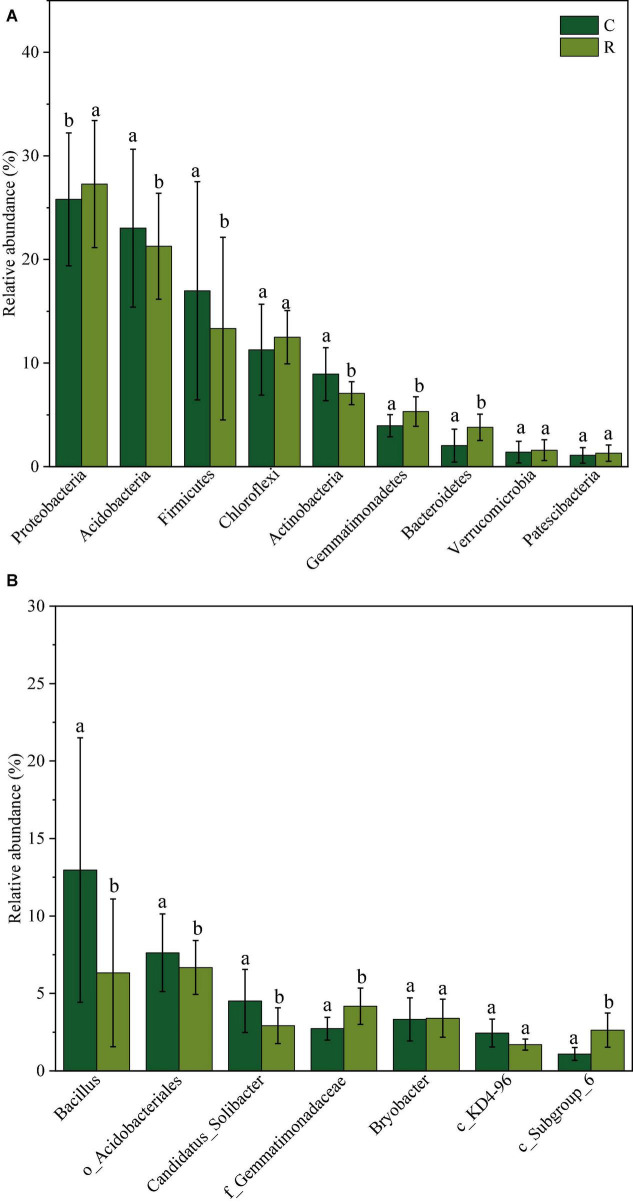
Relative abundance of soil bacterial phyla **(A)** and genus **(B)** in tobacco continuous and tobacco-rice rotation cropping soil.

Bacterial community composition in tobacco continuous and rotation cropping soil systems is depicted in Figure 2. At the phylum level (Figure 2A), Proteobacteria, Acidobacteria, Firmicutes, Chloroflexi, Actinobacteria, Gemmatimonadetes, Bacteroidetes, Verrucomicrobia, and Patescibacteria were the dominant phyla with the relative abundance over 1%. Among them, Proteobacteria showed the highest relative abundance, which increased from 25.80 to 27.28% in continuous cropping soil compared to rotation cropping soil. The relative abundance of Gemmatimonadetes and Bacteroidetes also increased from 3.95 to 5.32% and from 2.02 to 3.78%, respectively. However, the relative abundance of Acidobacteria, Firmicutes, and Actinobacteria decreased significantly in continuous cropping soil compared to that observed in rotation cropping soil. At the genus level (Figure 2B), *Bacillus* sp. was dominant (10%), but their average relative abundance decreased from 12.96% in continuous tobacco soil to 6.33% in tobacco-rice rotation soil, indicating that *Bacillus* sp. could not adapt to the changing soil environmental conditions. In addition, several relatively abundant genera, such as *o_Acidobacteriales* and *Candidatus_Solibacter*, also presented a decreasing tendency from7.53 to 6.48% and from 4.44 to 2.75%, respectively, in continuous cropping soil compared to rotation cropping soil. In contrast, the population of *f_Gemmatimonadaceae* and *c_Subgroup_6* slightly increased in rotation cropping soil.

#### Beta-diversity analysis of bacterial community structure

**FIGURE 3:**
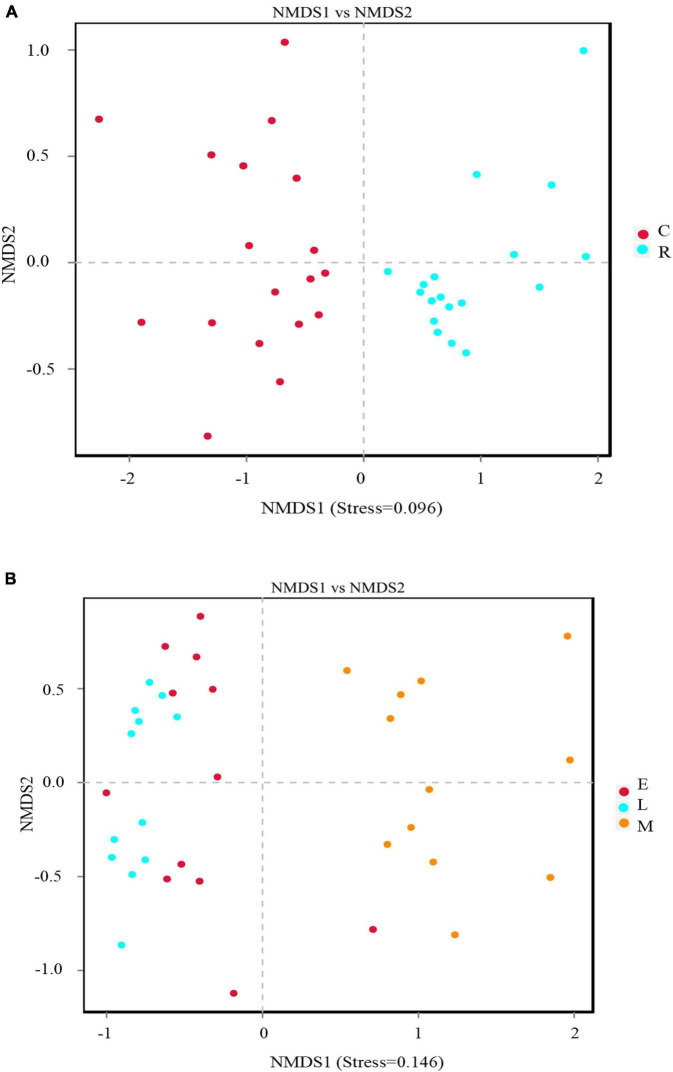
Non-metric multidimensional scaling (NMDS) analysis based on binary_jaccard method of rhizosphere soil bacterial community composition grouped according to cropping method **(A)** and growth stage **(B)**.

Non-metric multidimensional scaling (NMDS) analysis was used to visualize the dissimilarities in community composition between the samples of different cropping systems. As a measure of beta-diversity, NMDS plots in this study were obtained based on the Bray-Curtis method. Apparently, the soil samples subjected to different cropping systems were divided into two groups (Figure 3A) with stress of 0.096, indicating that the cropping method influenced the rhizosphere soil bacterial community composition significantly. Moreover, the bacterial communities of different tobacco growth stages were also clustered into two groups distinctly (Figure 3B). The samples of tobacco prosperous long-term stage and topping stage grouped together, while the samples of tobacco mature stage grouped separately from others, suggesting the different bacterial community composition. Moreover, the stress value of the soil bacterial community spatial ordination for different growth stages was 0.146, which suggested that the NMDS results had certain reliability, as the stress value was less than 0.2 (Taguchi and Oono, 2005).

### Biomarker analysis

**FIGURE 4:**
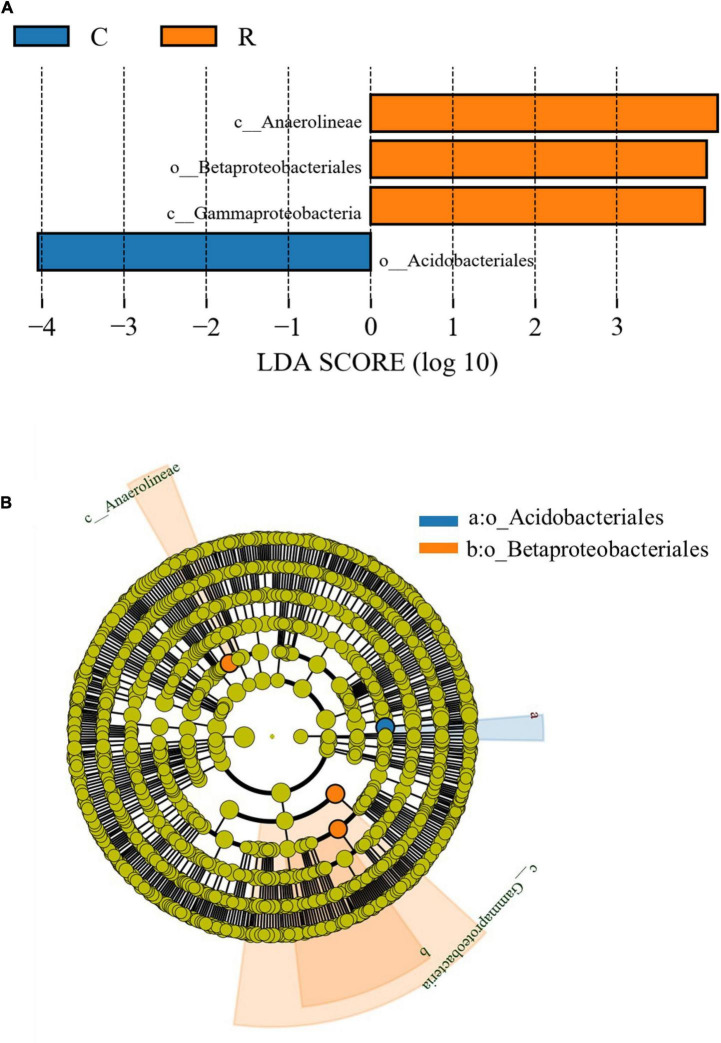
Comparative linear discriminant analysis (LDA) effect size (LEfSe) results of soil bacterial abundance of tobacco continuous and tobacco-rice rotation cropping systems. **(A)** Identified biomarkers ranked by the effect size in different cropping systems and **(B)** LEfSe taxonomic cladogram.

There were significant differences in the bacterial community composition between the two cropping systems (Figure 4). Four taxa with significantly different relative abundances were presented among the various sample groups according to the LEfSe pipeline (LDA > 3 or LDA < -3, *p* < 0.05). LEfSe has been widely used to find biomarkers among different sample groups associated with rhizospheric bacteria showing differences in their relative abundance (Mukhtar et al., 2021). In particular, in this study, three taxa, including *c_Anaerolineae*, *o_Betaproteobacteriales*, and *c_Gammaprotebacteria*, were enriched in the rotation cropping system, while one taxon (*o_Acidobacteriales*) was enriched in the continuous cropping system. These significantly different biomarkers may be important sources of differences in the microbial community structure in different cropping systems.

## Discussion

### Influence of cropping system on soil physicochemical properties

Tobacco planting significantly changed the soil physicochemical properties and resulted in an increase in soil fertility (e.g., SOC, TN, AP, and AK) and mineral content (e.g., exchangeable Ca/Mg). These results might be attributed to the interactions between tobacco and rhizosphere microorganisms, which released some root exudates and improved the soil microenvironment (Jin et al., 2022). In addition, tobacco planting improved the acidity of the soil by elevating the concentration of alkaline elements, such as exchangeable Ca/Mg (Han et al., 2019). Specifically, tobacco continuous cropping presented better effects on improving the soil environment than the tobacco-rice rotation cropping system. This observation was contradictory to other studies which indicated that the rotation cropping system was beneficial in enhancing soil fertility (Malobane et al., 2020; Zhang et al., 2022). This result can be ascribed to the amount of debris remaining in the field after each crop in a continuous cropping system, even after removing the tobacco stalks (Triberti et al., 2016). Chlorine is an essential nutrient element in the process of tobacco growth, and the chlorine content of tobacco leaves is an important factor that affects its quality (Qiang et al., 2011). The content of chlorine in the soil directly affects the level of chlorine in the tobacco leaves. The tobacco planting soil is divided into five grades based on the presence of the water-soluble content: very low (< 5.00 mg/kg), low (5.00–10.00 mg/kg), suitable (10.01–30.00 mg/kg), high (30.01–40.00 mg/kg), and very high (> 40.00 mg/kg) (Karaivazoglou et al., 2005). The content of water-soluble Cl in the tobacco continuous and tobacco-rice rotation cropping systems was lower than that noticed in the original soil, which can be attributed to the uptake of tobacco.

### Changes in tobacco rhizosphere bacterial community

Based on the results of alpha-diversity analysis, rotation cropping significantly increased the richness of bacterial species compared to the continuous cropping system, which was indicated by a significantly higher number of observed OTUs and a higher Chao 1 index (Figure 1). It might be because the composition of rhizosphere bacteria of tobacco and rice in the rotation cropping system was different, thus increasing the species richness (Liu et al., 2020). Moreover, the Shannon and Simpson indexes also presented a significantly increasing trend, suggesting that the diversity of rhizosphere bacteria in the rotation cropping system soil increased (Jin et al., 2022). Apparently, the observed OTU number, Chao1, Shannon, and Simpson indexes in the tobacco and rice rotation soils were higher than that in tobacco continuous soil, which demonstrates that the rhizosphere bacteria in the rotation cropping system soil showed increased richness and diversity. The results were in accordance with previous research that indicated soil bacterial community generally declined with long-term monocropping (Chen et al., 2020). In comparison, bacterial richness and diversity showed no changes between different tobacco growth periods in the continuous cropping system, as there was no significant difference in the observed OTU number, Chao1, Shannon, and Simpson indexes, indicating that the plant type was the major factor that determined the rhizosphere bacterial community. However, in the rotation cropping system, both bacterial richness and diversity decreased during the mature period in plants. The changed soil properties in the mature period, such as pH and depleted available nutrients (SOC and AP), might have resulted in this observation (Ding et al., 2017). In addition, many studies confirmed the resource competition theory that microbial diversity is the highest under moderate resource limitation, while it decreased when the environmental resources were limited (Graham and Duda, 2011; Wei et al., 2021).

According to the analysis of bacterial community composition at the phylum level, Proteobacteria, Acidobacteria, Firmicutes, Chloroflexi, Actinobacteria, Gemmatimonadetes, Bacteroidetes, Verrucomicrobia, and Patescibacteria were the dominant phyla, but their relative abundances changed obviously in different cropping systems. The continuous cropping system makes the soil bacterial community structure simple, which was similar to that reported by Chen et al. (2020), who showed that the bacteria exhibit apparent changing biodiversity along with the succession of crops. The increased bacterial population in rotation cropping soil can be attributed to their adaptability to a new microenvironment (Yin et al., 2010). However, the modification of the soil bacterial community structure could lead to the sickness of soil (Chen et al., 2014). At the genus level, *Bacillus* was the most abundant bacteria, and they are found to be beneficial to plant growth (Gomaa, 2012). However, their relative abundance decreased significantly in the rotation cropping system, which might affect tobacco growth and yield. In addition, the genera related to the degradation of potential soil allelochemicals (Wang et al., 2019a), such as *o_Acidobacteriales* and *Candidatus_Solibacter*, also presented a decreasing tendency in the rotation cropping system. This finding indicated that the bacterial community structure was significantly different between tobacco continuous and tobacco-rice rotation cropping systems. Moreover, the bacterial community composition of the mature period was different from the prosperous long-term and topping stages. The result was contradictory to previous research that indicated the peanut growth stage had less influence on bacterial communities (Chen et al., 2020). Here, the different observations might be explained by the plant variety (Liu et al., 2020).

The indicator genera of the bacterial community composition of the tobacco-rice rotation cropping system were *c_Anaerolineae*, *o_Betaproteobacteriales*, and *c_Gammaprotebacteria*. The genus *c_Anaerolineae* is predominantly found in the anaerobic environment and participates in denitrification, which drives the nitrogen cycle (Li et al., 2020). The paddy soil is beneficial for *c_Anaerolineae* enrichment. The genera *o_Betaproteobacteriales*, and *c_Gammaprotebacteria* belong to Proteobacteria, and their relative abundance was related to the relatively higher soil pH, and lower content of SOC and TN (Li et al., 2014). Thus, the characteristics of the rotation cropping soil (see section “Soil characteristics”) were the major drivers for the enrichment of *o_Betaproteobacteriales* and *c_Gammaprotebacteria*. *o_Acidobacteriales* is the indicator genus in the tobacco continuous cropping system. It is a Gram-positive bacteria and well adapted to the acidic environment and heavy metals in the soil (Tang et al., 2020). These significantly different biomarkers may be important sources of differences in the microbial community structure in different cropping systems. These results also indicated that different cropping systems changed the soil properties, which further led to variation in the microbial diversity.

## Conclusion

We investigated the soil properties and changes in the rhizosphere bacterial community structure in tobacco continuous and tobacco-rice rotation cropping systems. Significant increases in soil SOC, TN, and the content of other nutrients (e.g., AP and AK) were observed in tobacco continuous cropping compared to tobacco-rice rotation cropping. However, the alpha-diversity indexes, such as Chao 1, Simpson, and Shannon, in the continuous cropping system were significantly lower (*p* < 0.05) than those observed in the rotation cropping system, implying a loss in general microbial richness and evenness. The results of NMDS indicated that the cropping systems affected the tobacco rhizosphere bacterial composition significantly, and continuous cropping made the soil bacterial community structure simple. The rhizosphere bacterial community structure showed no significant changes in the tobacco prosperous long-term stage and topping stage, but it changed significantly in the mature stage.

## Data availability statement

The data presented in the study are deposited in the NCBI repository, accession number PRJNA866190.

## Author contributions

PW: conceptualization, methodology, investigation, validation, data curation, visualization, writing—original draft, and writing—review and editing. SY: formal analysis, visualization, and writing—review and editing. WZ: investigation and validation. XX, ML, TR, and LG: conceptualization and methodology. ZZ: conceptualization and funding acquisition. All authors contributed to the article and approved the submitted version.
